# Difficult-to-Treat Depression: Effectiveness, Functionality, and Quality of Life—Preliminary Results from a Real-World Prospective Cohort under Esketamine Treatment in Spain after 3-Month Follow-Up

**DOI:** 10.1192/j.eurpsy.2025.347

**Published:** 2025-08-26

**Authors:** M. Gomez Revuelta, G. Cortez Astudillo, J. Sastre Yáñez, D. Gutiérrez Hormaechea, P. Fuentes Pérez, C. Ovejas Catalán, J. L. Victores Barcia, M. Fernandez Rodriguez, N. Vargas Berni

**Affiliations:** 1 Psychiatry, IDIVAL, Santander; 2 Sierrallana Hospital, Torrelavega; 3 IDIVAL; 4 Hospital Universitario Marqués de Valdecilla, Santander; 5 HU Reina Sofía, Cordoba, Spain

## Abstract

**Introduction:**

Major depressive disorder (MDD) is one of the most prevalent and disabling mental health conditions globally. Approximately one-third to half of MDD patients, suffer from difficult-to-treat depression (DTD), a condition marked by persistent symptoms that do not respond to multiple standard treatments. DTD is often associated with comorbid physical and psychiatric conditions leading to chronic disability and a reduced quality of life. The complexity of DTD poses a considerable challenge in clinical practice, underscoring the need for innovative treatment options.

**Objectives:**

Despite the demonstrated efficacy of esketamine in controlled clinical trials, real-world evidence is limited. This study aimed to address this gap by assessing the effectiveness of esketamine in routine clinical practice in DTD patients.

**Methods:**

This prospective, naturalistic, open-label, observational study was conducted at Marqués de Valdecilla University Hospital in Cantabria, Spain. It included 33 patients diagnosed with DTD, comprising both unipolar and bipolar depression, as well as persistent depressive disorder (see table 1). Esketamine was administered intranasally in doses ranging from 56 mg to 84 mg, across three phases: induction, consolidation, and maintenance. Treatment effectiveness was measured using the MADRS, Clinical Anxiety Scale (CAS), and Clinical Global Impression (CGI). Functionality and quality of life were assessed with the Brief Functioning Scale (FAST) and the EQoL-5D (ESH). Assessments were conducted at baseline, 1-month, and 3-month. Data analysis was performed using SPSS v26, with repeated measures ANOVA and Pearson’s χ² tests employed to evaluate changes over time.

**Results:**

The final analysis included 33 patients with DTD and long-lasting current MDD episodes (table 2). Baseline MADRS scores indicated severe depression (39.12 ± 6). Significant reductions in MADRS scores were observed at both one month (21.61 ± 11.15) and 3-month (19.70 ± 11.65) compared to baseline. At 3-month, 54.5% of patients achieved a ≥50% reduction in MADRS scores, and 30.3% reached remission (MADRS < 12). Similar improvements were seen in anxiety (CAS) and health status (ESH) scores, with significant reductions noted over time. However, functional improvements (FAST) were not statistically significant (see table 3).

**Conclusions:**

Esketamine demonstrated substantial effectiveness in reducing both depressive and anxiety symptoms in DTD patients over three months. More than half of the patients achieved a significant reduction in depression severity, with nearly a third reaching remission. The presence of late responders suggests that esketamine may benefit those initially unresponsive to treatment. These findings support esketamine as a valuable therapeutic option for DTD in real-world clinical settings.

**Disclosure of Interest:**

None Declared

